# Definitive concurrent chemoradiotherapy with docetaxel plus cisplatin versus 5-fluorouracil plus cisplatin in patients with esophageal squamous cell carcinoma: long-term follow-up results of a phase II randomized controlled trial

**DOI:** 10.1186/s13014-023-02339-9

**Published:** 2023-09-12

**Authors:** Hui Jiang, Kanjiebubi Makelike, Baoqing Chen, Mian Xi, Qiaoqiao Li, Yonghong Hu, Yujia Zhu

**Affiliations:** Department of Radiation Oncology, State Key Laboratory of Oncology in South China, Collaborative Innovation Center for Cancer Medicine, Sun Yat-sen University Cancer Center, 651 Dongfeng East Road, Guangzhou, 510060 Guangdong P.R. China

**Keywords:** Esophageal cancer, Chemotherapy, Radiotherapy, Esophagus, Recurrence, Salvage treatment

## Abstract

**Background:**

Definitive radiotherapy plus concurrent chemotherapy has been a standard treatment for esophagus patients who are unfit to undergo surgery. However, there are a variety of concurrent chemotherapy regimens with varying efficacy. In this phase II prospective study, we compared the efficacy and toxicity of DP (docetaxel and cisplatin) and PF (cisplatin and 5-fluorouracil) regimens with concurrent chemoradiotherapy (CCRT) in patients with esophageal squamous cell carcinoma (ESCC) and analyzed the 5-year overall survival (OS) and progression free survival (PFS). We also summarized the salvage treatments and late toxicities.

**Methods:**

We enrolled 86 patients with clinical stage II-IVA from the Sun Yat-sen University Cancer Center. The patients were divided into two groups: PF group (41) and DP group (45). Statistics were analyzed using SPSS version 19.0.

**Results:**

The 5-year OS rates were 62.9% ± 7.6% in PF group, and 52.7% ± 7.5% in DP group (P = 0.131), respectively. The 5-year PFS rates were 43.9% ± 7.8% for PF group, and 40.0% ± 7.3% for DP group (P = 0.398), respectively. Sixteen patients in the DP group and thirteen in the PF group received salvage treatment. For those patients with local residual or local recurrent disease, the median survival time after salvage treatment was 13.5 months and the 1, 2, and 3-year survival rates were 79.0%, 50.3%, and 43.1%, respectively. For all patients, thirteen (15.1%) had Grade 2 late cardiac toxicities. One patient had Grade 2 pleural effusion and required diuretic. Most patients with pneumonia are mild, and only one patient in PF group had Grade 2 pneumonia. One patient in the DP group developed tracheoesophageal fistula.

**Conclusions:**

The 5-year follow-up confirmed that definitive CCRT with the DP regimen did not improve the treatment response, OS, or PFS in patients with ESCC compared to the PF regimen. The PF regimen remains the standard regimen for definitive CCRT for patients with locally advanced ESCC. Long-term follow-up also suggested that appropriate and active salvage treatment has a survival benefit for some patients, and late cardiopulmonary toxicities should be noticed during follow-up.

**Trial registration:**

The trial was registered at https://clinicaltrials.gov (ClinicalTrials.gov Identifier: NCT 02969473, October 2010).

## Introduction

Esophageal carcinoma (EC) is the ninth most prevalent cancer and the sixth-highest cancer-related mortality in the world [1]. The incidence of EC in China is the highest in the world, accounting for over 50% of mortality and morbidity. About 90% of EC patients in East Asia have esophageal squamous cell carcinoma (ESCC) [2]. As the standard treatment option for patients not eligible for surgery, definitive radiotherapy with concurrent chemotherapy has been established [3–7]. For EC patients, 5-fluorouracil plus cisplatin (CDDP) (PF regimen) has been the most widely used chemotherapy regimen since the 1980s [3–8]. However, the toxicity and the survival of definitive concurrent chemoradiotherapy (CCRT) with cisplatin plus fluorouracil regimen have hitherto not been satisfactory. It was associated with 42.5% grade 3 acute toxicity, 25.5% grade 3 late toxicity, and 26.5% 5-year overall survival (OS) [6]. A great deal of effort has been invested into improving the OS and locoregional control through the combination of different chemotherapy medications. Unfortunately, when compared to the PF regimen, many trials have not shown a statistically significant benefit [3, 9, 10].

In some preclinical studies, it has been improved that, as a kind of semi-synthetic taxane, docetaxel was a radiation sensitizer [11–13]. Clinical studies have shown that docetaxel has notable therapeutic effect on head and neck squamous cell carcinoma [14, 15]. According to some studies, docetaxel and cisplatin chemotherapy administered concurrently with radiotherapy was highly effective in treating unresectable localized ESCC with tolerable toxicities, which has been hailed as promising [16, 17]. Therefore, in order to provide strong evidence for the efficacy of the DP regimen, it is necessary to obtain high-quality data from prospective randomized controlled trials.

This phase II prospective randomized study compared the efficacy and toxicity of DP and PF regimens with CCRT in ESCC patients. From October 2010 to March 2015, 86 patients from Sun Yat-sen University Cancer Center (SYSUCC) participated in this study. The preliminary outcomes were published in 2017 after 24 months of follow-up (median 25.1 months) [18]. We additionally investigated the consistency of long-term results with our earlier findings and analyzed the 5-year OS and progression free survival (PFS). We also summarized the late adverse events and post-relapse treatments of some patients, which provides a reference for clinicians.

## Materials and methods

### Patients

Patients’ inclusion criteria and the procedures of this prospective, single-center, randomized phase II trial have previously been reported. Eligible patients were aged between 18 and 70 years; had adequate bone marrow, hepatic, and renal function; a Karnofsky performance status (KPS) score ≥ 70; and had no history of other malignancies. Only patients with locally advanced (clinical stage II to IVA, including metastatic celiac or cervical nodes, according to the 6th edition of the American Joint Committee on Cancer [AJCC] staging system for esophagus cancer), histologically proven, and potentially curable ESCC, were eligible for inclusion. Primary exclusion criteria were the presence of distant metastasis (except for those confined to the celiac or cervical nodes) before treatment, a history of hypersensitivity to CDDP, 5-fluorouracil and docetaxel, and patients who were pregnant or breastfeeding. Our study protocol was reviewed by the SYSUCC Ethics Committee and approved. All patients gave their written informed consent to participate in the study.

### Randomization

After confirming the eligibility criteria, using a computerized randomization program, patients were divided into either the PF (41 patients) or DP (45 patients) treatment group. Trial registration was completed with the US National Institute of Health (ClinicalTrials. gov, Identifier NCT 02969473).

### Procedures

All patients underwent pretreatment evaluations, including a complete medical history and physical examination; complete blood count and chemistry profile of the serum; urine test; test of pulmonary function; electrocardiogram; examination of the barium swallow; neck, chest, and upper abdomen computed tomography (CT) scan with contrast; and ultrasonography by endoscopy. The patient underwent bronchoscopy if the diagnosis of bronchial invasion was suspected. Radionuclide bone scans and co-registered 18 F-labeled fluoro-2-deoxy-D-glucose positron emission tomography (PET/CT) scans were also performed when clinically indicated.

**Fig. 1:**
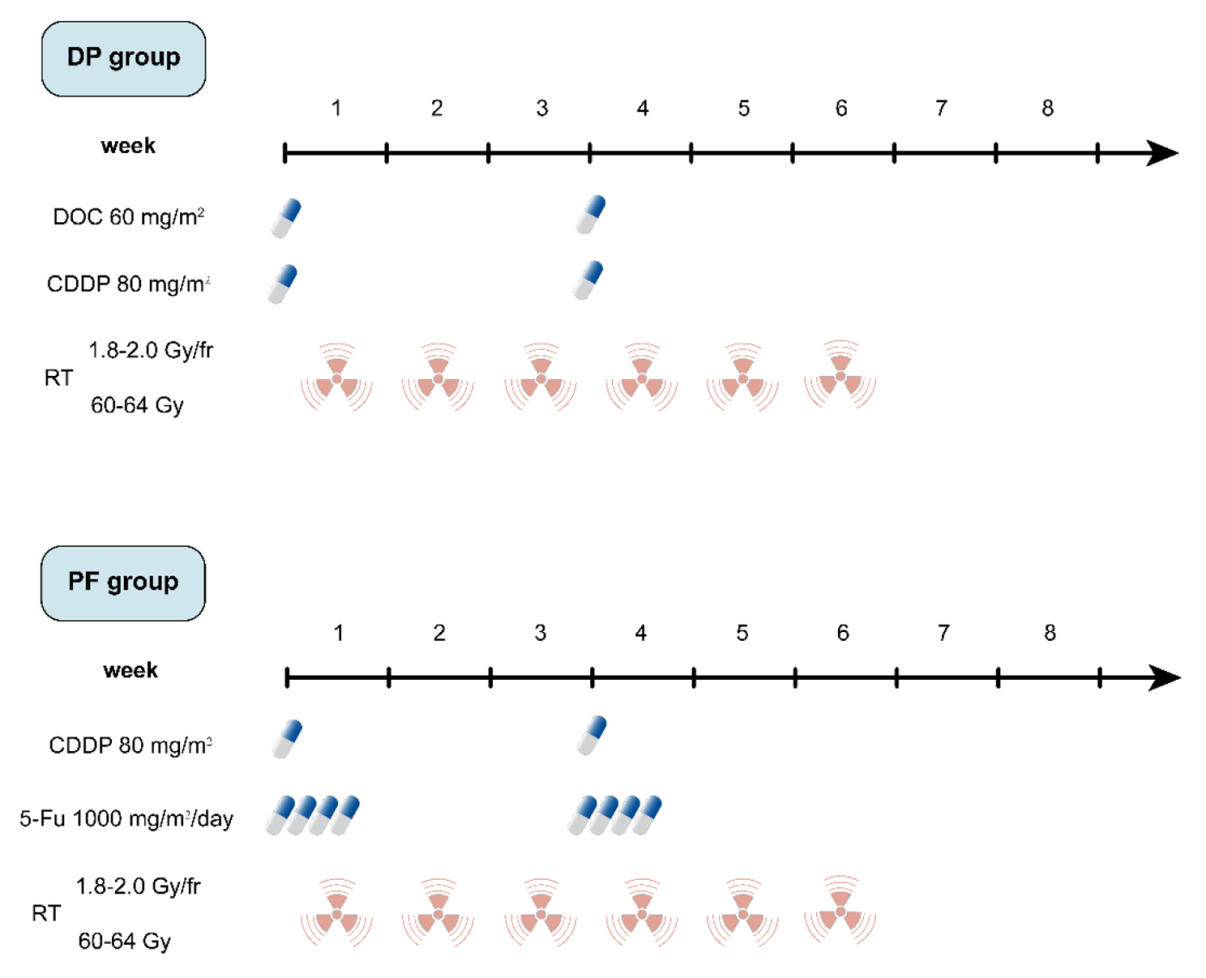
Treatment schedule of concurrent chemoradiotherapy in the DP group CDDP: cisplatin; DOC: docetaxel; DP: docetaxel plus cisplatin; fr: fraction; PF: cisplatin plus 5-FU

The treatment plan is illustrated in Fig. 1. Two cycles of chemotherapy were administered with CCRT to all patients. For patients assigned to receive CCRT with the DP regimen, docetaxel (60 mg/m^2^ delivered on day 1) and cisplatin (80 mg/m^2^ delivered on day 1) were intravenously administered at 3-week intervals. For patients assigned to receive CCRT with the PF regimen, 5-fluorouracil (1000 mg/m^2^ continuous infusion over 24 h daily on days 1–4) and cisplatin (80 mg/m^2^ delivered on day 1) were intravenously administered at 3-week intervals. Linear accelerators with a power of 6-MV or 8-MV were used to treat all patients. All patients received conventional radiotherapy at 1.8-2.0 Gy per fraction and five fractions per week. The total concurrent radiation dose was 60–64 Gy, from the first day of the first chemotherapy cycle onwards. Gross tumor volume (GTV) encompassed primary mass and metastatic regional lymph nodes observed on imaging examinations. Emerging evidence has demonstrated that involved field irradiation (IFI) is an acceptable treatment for locally advanced ESCC. In line with this notion, our trial employed IFI as well. The clinical target volume (CTV) included the primary tumour plus a 3.0-cm craniocaudal margin and a 1.0-cm margin in other directions, as well as the metastatic lymph nodes plus a 1.0-cm margin. The planning target volume (PTV1) included the GTV with a 5-mm margin in all directions, and the PTV2 generally included the CTV with a 5-mm margin in all directions. The prescribed dose was 60 to 64 Gy for PTV1 and 50 Gy for PTV2. The patients received either intensity-modulated radiation therapy (IMRT) or three-dimensional conformal radiotherapy (3D-CRT). We use the Monacle planning system (Monacle version 5.11, ELEKTA, US) to calculate the IMRT treatment plans, while 3D-CRT treatment plans were calculated using the Pinnacle planning system (Pinnacle3 version 8.0 m; Philips Medical Systems, Fitchburg, WI, USA). In some cases of severe hematologic or non-hematologic toxicity, dose adjustment was performed in the subsequent chemotherapy cycle. The following section outlines commonly implemented adjustments in chemotherapeutic drug regimens. In the presence of grade 3 thrombocytopenia, febrile grade 3 neutropenia, grade 4 neutropenia, or grade 3 non-hematologic toxicities, drugs are reduced to 75% of the initial dose. In cases of grade 4 thrombocytopenia, febrile grade 4 neutropenia, or grade 4 non-hematologic toxicities, drugs are adjusted to 50% of the original dose. Following dose reduction, if the same grade or higher toxicity recurs, drugs are further adjusted to 50% of the initial dose. In the event of grade 4 toxicity occurring subsequent to dose adjustment for grade 4, treatment is discontinued. The first follow-up took place in the fifth or sixth week after the end of treatment, and every three months thereafter for up to two years. It was planned to follow up every six months for up to five years or when clinically necessary.

### Outcomes

The primary endpoint was the OS, which was defined as the time from randomization to death from any cause or the last follow-up date. Secondary endpoints included treatment response rate, PFS, patterns of treatment failure, completion rate of the protocol, and late toxicities associated with the treatment. PFS meant the time from randomization to progression, relapse, death from any cause, or last follow-up date.

### Statistical analysis

Intention-to-treat analysis was used to analyze the data. The sample size was calculated using Power and Sample Size Calculations software (Version 3.0, 2009, USA). On the basis of previous study, the current study was estimated to detect an improvement in the median OS of 35 months for the DP group to 20 months for the PF group with a two-sided test with alpha level 0.05 and 80% statistical power. An estimated sample of 182 participants (91 per group) was required. Because recruitment in this trial was slower than expected, we performed an interim analysis in March 2015, after 86 patients had been enrolled. The Kaplan-Meier method was used to estimate survival, and the log-rank test was used to determine significance. It was considered significant only if the P-value was < 0.05 on two sides. Comparing baseline characteristics of the treatment groups was done using the t-test or Mann-Whitney U-test for continuous variables, and the Fisher’s exact test for categorical variables. Statistics were analyzed using SPSS version 19.0.

## Results

**Fig. 2:**
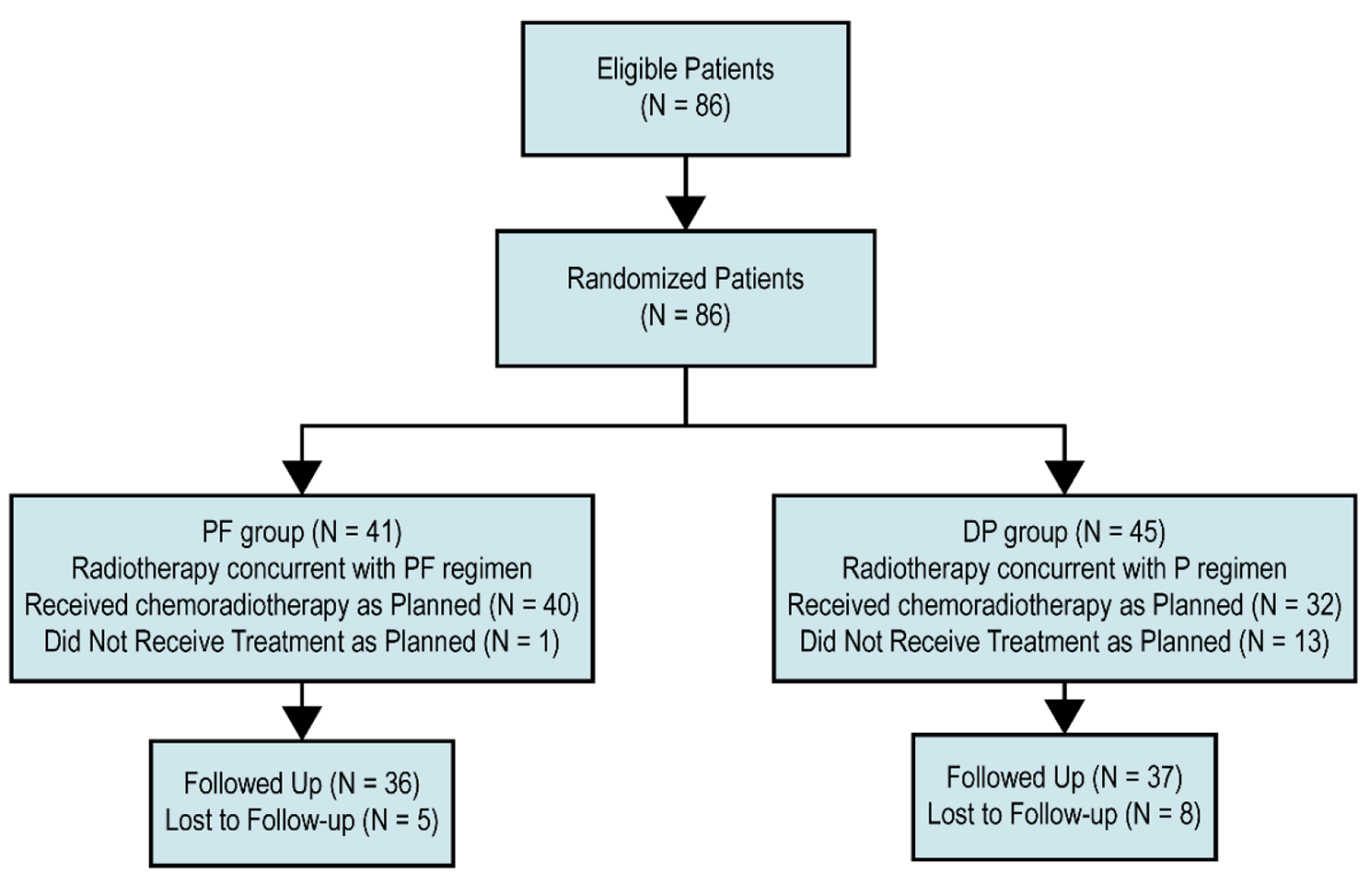
Trial profile DP: docetaxel plus cisplatin; PF: cisplatin plus 5-FU

From 2010 to 2015, we enrolled 86 patients in this trial, PF group had 41 participants and DP group had 45 (Fig. 2). Due to the slow enrollment, we terminate early. There was a good balance between the two groups in terms of demographics and characteristics of tumor (Table 1). The treatment compliance details for the patients are listed in Table 2. All patients in the two groups completed radiotherapy as planned, with 35 (85.4%) receiving IMRT and 6 (14.6%) patients receiving 3D-CRT in the PF group versus 33 (73.3%) receiving IMRT and 12 (26.7%) patients receiving 3D-CRT in the DP group (P = 0.171). As for chemotherapy, PF group had 40 (96.7%) patients complete full doses for two cycles, while the DP group had 32 (71.1%) patients complete full doses for two cycles (P = 0.002). Due to the early treatment-induced toxicity, one (2.4% of the PF patients) required an altered chemotherapy regimen during the second cycle. However, in the DP patients, eight (17.8%) received dose reduction, three (6.7%) received an altered regimen, and two (4.4%) canceled chemotherapy during the second cycle. According to the evaluation criteria for clinical efficacy, in the PF group, 12 patients obtained complete remission (CR), 24 patients achieved partial remission (PR), four patients experienced stable disease (SD), and one suffered progressive disease (PD). In the DP group, there were 14, 24, 5, and 2 patients with CR, PR, SD, and PD, respectively. The overall response rate (ORR: CR + PR) was 87.8% in the PF group and 84.4% in the DP group (P = 0.653) [18].

**Table 1:** Baseline clinical characteristics of the PF group and the DP group

Variable	PF group(N = 41) n%	DP group(N = 45) n%	P-value
Gender			
Male	29(70.7)	31(68.9)	0.853
Female	12(29.3)	14(31.1)	
Age (years)			
Median (range)	59(45–69)	58(40–73)	
< 60	25(61.0)	27(60.0)	0.926
≥ 60	16(39.0)	18(40.0)	
KPS			
90	33(85.0)	39(86.7)	0.370
80	8(19.5)	5(11.1)	
70	0(0)	1(2.2)	
BMI (kg/m²)			
Median (range)	21.5(15.7–30.8)	21.3(17.1–27.5)	
< 18.5	6(14.6)	4(8.9)	0.508
≥ 18.5	35(85.4)	41(91.1)	
CCI			
0	31(75.6)	31(68.9)	0.716
1	10(24.4)	13(28.9)	
3	0(0)	1(2.2)	
Smoking index			
0	15(36.6)	20(44.4)	0.755
> 0, ≤ 400	10(24.4)	10(22.2)	
> 400	16(39.0)	15(33.3)	
Drinking			
No	28(68.3)	30(66.7)	0.872
Yes	13(31.7)	15(33.3)	
Tumour location			
Cervical	7(17.1)	5(11.1)	0.729
Upper thoracic	13(31.7)	19(42.2)	
Middle thoracic	16(39.0)	15(33.3)	
Lower thoracic	3(7.3)	2(4.4)	
Multiple primary	2(4.9)	4(8.9)	
Tumour length (mm)			
Median (range)	55(10–150)	47(20–96)	
T stage*			
T1	1(2.4)	0(0)	0.191
T2	4(9.8)	10(22.2)	
T3	25(61.0)	28(62.2)	
T4	11(26.8)	7(15.6)	
N stage*			
N0	2(4.9)	3(6.7)	1.000
N1	39(95.1)	42(93.3)	
M stage*			
M0	30(73.2)	30(66.7)	0.512
M1a	11(26.8)	15(33.3)	
TNM stage*			
IIA, IIB	4(9.8)	9(20.0)	0.236
III	26(63.4)	21(46.7)	
IVA	11(26.8)	15(33.3)	

Abbreviations: BMI = body mass index; CCI = Charlson comorbidity index; KPS = Karnofsky performance status; PF = cisplatin + 5-Fu; DP = docetaxel + cisplatin; *TNM stage was assessed according to the 6th edition of the American Joint Commission on Cancer (AJCC) staging system

**Table 2:** Treatment information for the PF group and DP group

Variable	PF group (N = 41)	DP group(N = 45)	P-value
Radiotherapy techniques			
3D-CRT	6 (14.6)	12 (26.7)	0.171
IMRT	35 (85.4)	33 (73.3)	
Radiation dose (Gy)			
Median (range)	60.0 (56.0–64.0)	60.0 (55.8–64.0)	
Treatment compliance			
As planned	40 (97.6)	32 (71.1)	0.002
s cycle of chemotherapy reduced	0 (0)	8 (17.8)	
Second cycle of chemotherapy changed	1 (2.4)	3 (6.7)	
Second cycle of chemotherapy cancelled	0 (0)	2 (4.4)	

Abbreviations: 3D-CRT = three-dimensional conformal radiotherapy; IMRT = intensity-modulated radiotherapy

**Fig. 3:**
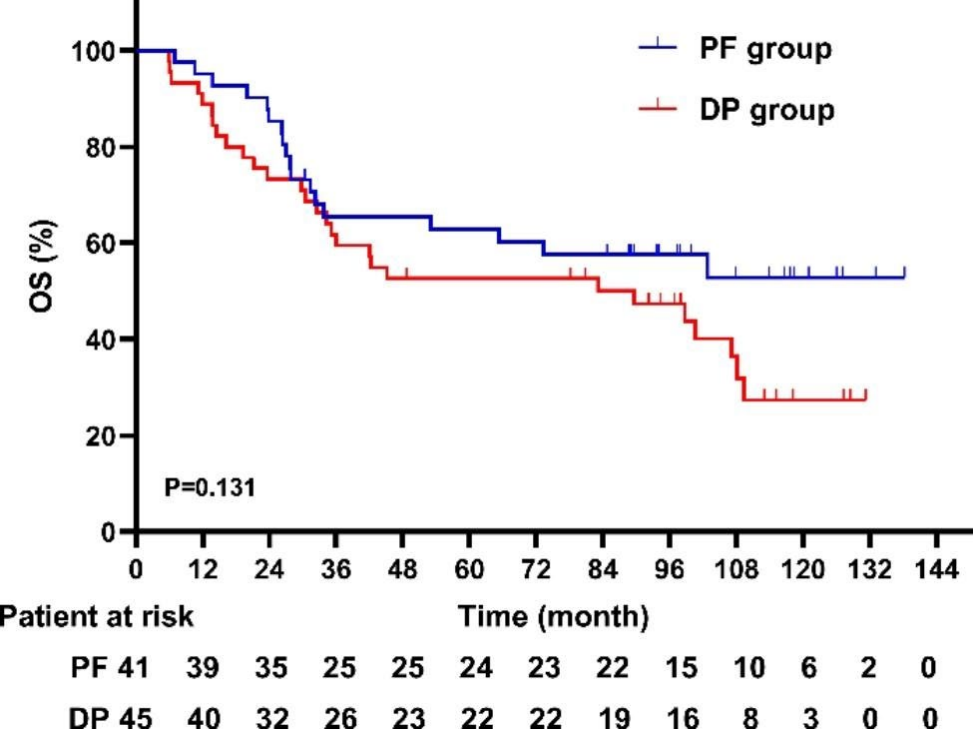
Overall survival of the DP group and the PF group DP: docetaxel plus cisplatin; OS: overall survival; PF: cisplatin plus 5-FU

**Fig. 4:**
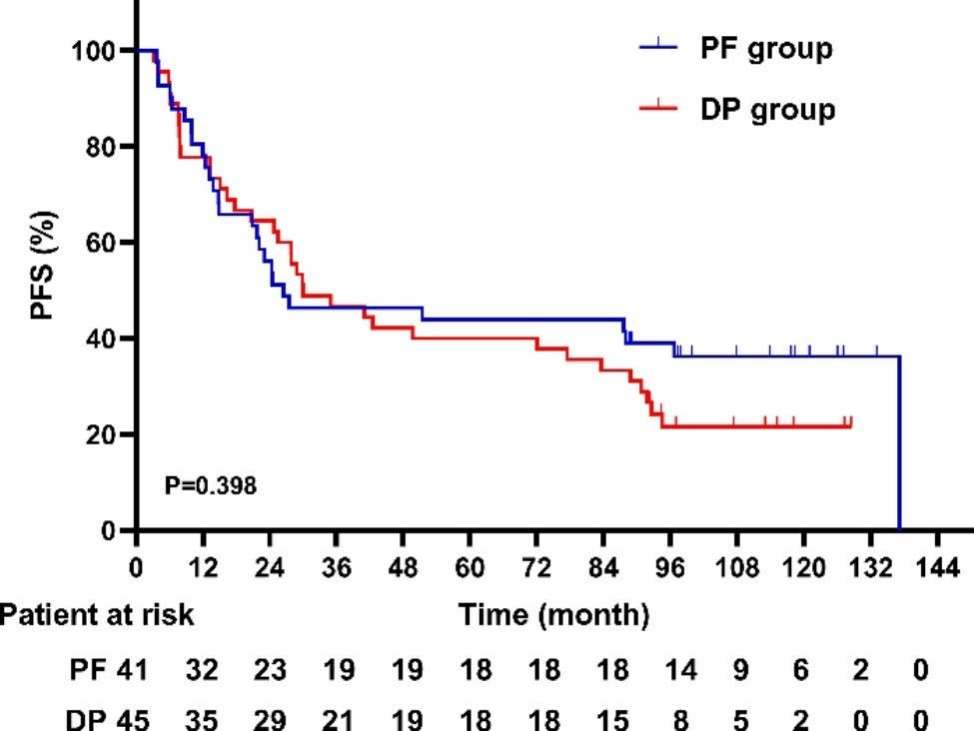
Progression-free survival of the DP group and the PF group DP: docetaxel plus cisplatin; PF: cisplatin plus 5-FU; PFS: progression-free survival

As of the time of this analysis, the median follow-up period for the surviving patients was 107 months (range 98–116). Of the 86 analyzed patients, 46 deaths (53.5%) were recorded at the final analysis (18 [43.9%] of 41 patients in the PF group and 28 [62.2%] of 45 patients in the DP group). The 5-year OS rates were 62.9% ± 7.6% in the PF group, and 52.7% ± 7.5% in the DP group. There was no significant difference in 5-year OS (62.9% vs. 52.7%, P = 0.131) between the PF and the DP group, respectively (Fig. 3). The 5-year PFS rates were 43.9% ± 7.8% for the PF group, and 40.0% ± 7.3% for the DP group (P = 0.398), respectively (Fig. 4). Twenty-four (27.9%) patients (10 (22.2%) in the DP group, and 14 (34.1%) in the PF group) were alive without disease progression at the time of analysis on March 5, 2022. The first failure patterns are listed in Table 3. Notably, more patients in the DP group had distant metastases at first failure than in the PF group (53.4% vs. 24.2%, P = 0.006), which is consistent with the previous study [18]. For patients who experienced disease progression, we summarized the treatment after progression. Sixteen patients in the DP group, received post-relapse therapy: one patient with simple distant metastasis underwent palliative radiotherapy; two patients with compound metastases received systemic chemotherapy; esophageal surgery was performed in one patient with simple local metastasis; one patient with simple local metastasis received chemoradiotherapy; and eleven patients with local or distant metastasis received chemotherapy alone. In the PF group, thirteen patients received post-relapse therapy: one patient with a simple distant metastasis underwent surgery at the metastatic site; one patient with compound metastasis underwent ablation plus chemotherapy; esophageal surgery was performed in two patients with simple local metastases; one patient with simple local metastasis received chemoradiotherapy; and eight patients with local or distant metastasis received chemotherapy alone. Notably, the salvage treatments and survival of some patients with local residual or local recurrent are summarized in Table 4. The median survival time after salvage treatment was 13.5 months in these patients. The 1, 2, 3-year OS rates were 79.0%, 50.3% and 43.1%. In particular, 3 (20%) of the 15 patients remained disease free after salvage treatment. The Common Terminology Criteria for Adverse Events version 3.0 (CTCAE 3.0) was used to evaluated patients’ adverse events. As for the early adverse events, compared to the PF group, the DP group was significantly more likely to suffer from hematological toxicity. Non-hematological toxicity was comparable between the two groups [18]. After long-term follow-up, late adverse events were also observed in some patients. Thirteen patients had Grade 2 late cardiac events, including heart failure and pericardial effusion (7 in the DP group, 6 in the PF group). One patient had Grade 2 pleural effusion, and required diuretics. Most patients with pneumonia were mild, and only one Grade 2 pneumonia was observed in PF group. One patient in the DP group developed tracheoesophageal fistula. No deaths due to the therapeutic toxicity were observed. In summary, the 1, 2, and 5-year OS rates were 93.7%, 86.2%, and 62.9%, respectively, in the PF group, and 87.3%, 69.1%, and 52.7%, respectively, in the DP group.

**Fig. 5:**
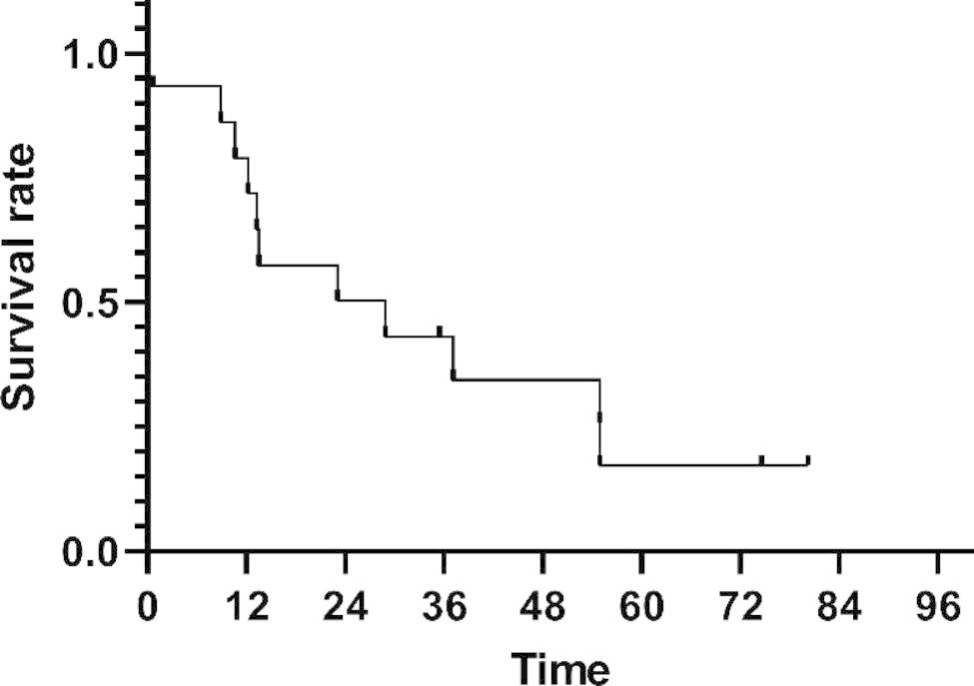
Kaplan-Meier survival plot for patients from initiation of different salvage treatments

**Table 3:** Location of disease at first treatment failure and patterns of failure

First failure	PF group (N = 41)		DP group (N = 45)	P-value
Location of disease at first treatment failure				
Local-regional only	17		12	
Distant only	4		12	
Local-regional and distant	6		12	
Patterns of failure				
Local-regional failure	23(56.1%)		24(53.4%)	0.797
Distant failure	10(24.2%)		24(53.4%)	0.006

**Table 4:** Regimens and results of salvage treatment for local recurrent or residual squamous cell carcinoma of the esophagus

Patient	Response for CCRT	Time to treatment failure (months)	Salvage treatment	Outcome	Duration ^#^ (months)	Survival from CCRT (months)
Residual cancer group						
1	SD	12.40	TP→FOLFIRI	Dead of disease	54.90	73.30
Local failure at primary site group						
2	PR	13.40	TP	Dead of disease	0.30	13.80
3	SD	77.43	TP + Radiotherapy	Dead of disease	28.93	108.20
4	PR	83.53	S-1 + Apatinib	Dead of disease	23.10	107.07
5	CR	20.77	Capecitabine	Dead of disease	13.27	34.17
6	CR	27.80	Surgery	Dead of disease	54.97	83.20
7	CR	14.90	TP + Surgery	Dead of disease	10.63	26.27
8	PR	137.23	S-1	Alive with disease	0.70	138.27
9	PR	10.07	Surgery	Dead of disease	12.17	23.63
10	PR	22.13	TP	Dead of disease	8.90	31.43
11	CR	27.43	Radiotherapy + S-1→TP + anti-PD-1 antibody→ Radiotherapy	Dead of disease	37.13	65.37
12	PR	6.40	Capecitabine	Dead of disease	13.50	20.03
13	PR	12.00	surgery	Alive, disease free	74.60	89.60
14	CR	51.40	TP + Radiotherapy	Alive, disease free	35.47	89.10
Metachronous multiple cancer group						
15	CR	49.73	Radiotherapy + Capecitabine	Alive, disease free	80.27	131.27

^#^ The duration was measured from the date of salvage treatment to the date of confirmation of death or the end of follow-up

Abbreviations: CCRT = concurrent chemoradiotherapy; SD = stable disease; PR = partial response; CR = complete response; TP = taxol + platinum; FOLFIRI = 5-fluorouracil + irinotecan + leucovorin; anti-PD-1 = anti-program death-1

## Discussion

For clinicians, esophageal carcinoma remains a challenging condition, with a poor prognosis and low survival rate. For fit patients with resectable esophageal carcinoma, the 5-year survival rate was only 25% after surgery [19]. Moreover, approximately 30% of the patients who underwent esophageal surgery, who were clinically considered to have resectable disease, had microscopically irradical resections performed [20–22]. Further evidence suggested that neoadjuvant concurrent chemoradiotherapy followed by surgery achieved a survival benefit [23]. Based on the aforementioned, some multi-center prospective trials were initiated to improve the survival benefit of neoadjuvant chemoradiotherapy or postoperative chemotherapy for patients with clinically resectable, locally advanced EC that offers a higher survival benefit over surgery alone, as demonstrated in the CROSS, JCOG9907, and NEOCRTEC5010 trials [24–28].

Nevertheless, at the initial diagnosis, approximately half of the patients are no longer eligible for surgery [29]. Therefore, it is particularly important to improve the survival of these patients. Over the past few decades, many studies have focused on finding surgery alternatives. Based on the results of the Radiation Therapy Oncology Group (RTOG) 8501 study, definitive radiotherapy concurrent with cisplatin plus fluorouracil has become one of the standard treatment regimens for locally advanced EC. Chemoradiotherapy for RTOG 8501 was the cisplatin plus fluorouracil plan, in which cisplatin 75 mg/m^2^ was administered on the first day of weeks 1, 5, 8, and 11, and a 1000 mg/m^2^ dose of fluorouracil was administered for the first 4 days of weeks 1, 5, 8, and 11 concurrent with radiation therapy (50 Gy in 25 fractions over 5 weeks). Although combined therapy proved to be superior to radiotherapy alone, the treatment toxicities and the survival were not satisfactory, with 42% grade 3 acute toxicity, 25% grade 3 late toxicity, and 26% 5-year OS [6]. Since then, a growing number of studies have evaluated the clinical outcomes of CCRT in patients with EC. The selected prospective studies and retrospective studies that have been updated are listed in Table 5 [10, 30–37].

**Table 5:** Studies on definitive radiotherapy with or without chemotherapy for oesophageal cancer patients

Study	Type	Pathology	Stage	N	Treatment (n)	Radiation dose (Gy)	Chemotherapy regimen	ORR	MST (month)	1 year OS	3 year OS	5 year OS
Herskovic A, 1992 [7]; al-Sarraf M, 1997 [8]; Cooper JS, 1999 [6]	Prospective Randomized (RTOG 85 − 01)	Sq/Ad	T1-3 N0-1 M0	123	RT alone (62)	64	/	/	9.3	34%	0%	0%
					RT + CT (PF) (61)	50	CDDP 75 mg/m^2^ d1 + 5-FU 1 g/m^2^/d d1-d4, Q4W	/	14.1	52%	30%	26%
Minsky BD, 1996 [30]; Minsky BD, 1999 [31];	Prospective (INT 0122)	Sq	T1-4 N0-1 M0	38	CT (PF) → RT + CT (PF) (38)	64.8	Neoadjuvant segment: CDDP 100 mg/m^2^ d1 + 5-FU 1 g/m^2^/d d1-d5, Q4W; Combined segment: CDDP 75 mg/m^2^ d1 + 5-FU 1 g/m^2^/d d1-d5, Q4W	55%	20	/	30%	20%
Atsushi Ohtsu, 1999 [32]	Prospective	Sq	T4/M1 LYM	54	RT + CT (PF) (54)	60	CDDP 40 mg/m^2^ d1, d8 + 5-FU 400 mg/m^2^/d d1-d5, d8-d12, Q5W	87%	9	41%	23%	/
Minsky BD, 2002 [5]	Prospective (INT 0123) (RTOG 94 − 05)	Sq/Ad	T1-4 N0-1 M0	218	RT + CT (PF) (109)	50.4	CDDP 75 mg/m^2^ d1 + 5-FU 1 g/m^2^/d d1-d4, Q4W	/	18.1	40%	/	/
					RT + CT (PF) (109)	64	CDDP 75 mg/m^2^ d1 + 5-FU 1 g/m^2^/d d1-d4, Q4W	/	13	31%	/	/
Ishida K, 2004 [33]	Prospective	Sq	T4/M1 LYM	60	RT + CT (PF) (60)	60	CDDP 70 mg/m^2^ d1 + 5-FU 700 mg/m^2^/d d1-d4, Q4W	68.3%	10	/	/	/
Ajani JA, 2008 [34]	Prospective randomized (RTOG 0113)	Sq/Ad	Localized	72	CT(TPF) → RT + CT (TF) (37)	50.4	/		28.7	75.7%		
					CT(TP) → RT + CT (TP) (35)	50.4	/		14.9	68.6%		
Li QQ, 2010 [17]	Retrospective	Sq	II - IV	59	RT + CT (DP) (59)	50–64	CDDP 80 mg/m^2^ d1 + Docetaxel 60 mg/m^2^ d1, Q3W	98.3%	22.6	/	36.7%	/
Kato K, 2011 [35]	Prospective (JCOG 9906)	Sq	II - III	74	RT + CT (PF) (74)	60	CDDP 40 mg/m^2^ d1, d8 + 5-FU 400 mg/m^2^/d d1-d5, d8-d12, Q5W	66.2%	29	/	44.7%	/
Nishimura Y, 2012 [36]	Prospective (KROSG0101/ JROSG021)	Sq/Ad	II - IV	91	RT + CT (PF) (46)	60	CDDP 70 mg/m^2^ d1 + 5-FU 700 mg/m^2^/d d1-d5, Q4W	/	/	/	/	35%
					RT + CT (PF) (45)	60	CDDP 7 mg/m^2^ d1-d5, d8-d12 + 5-FU 250 mg/m^2^/d d1-d14, Q4W	/	/	44%	22%	71%
Zhao T, 2012 [16]	Prospective	Sq	II - IV	90	RT + CT (PF) (45)	50.4	CDDP 75 mg/m^2^ d1 + 5-FU 250 mg/m^2^/d d1-d4, Q4W	53.3%	22.3	/	/	/
					RT + CT (DP) (45)	50.4	CDDP 75 mg/m^2^ d1 + Docetaxel 75 mg/m^2^ d1, Q4W	73.3%	43.2	/	/	/
Conroy T, 2014 [3]	Prospective (PRODIGE5/ ACCORD17)	Sq/Ad	I - IV	267	RT + CT (FOLFOX) (134)	50	/	67%	20.2		19.9%	
					RT + CT (PF) (133)	50	CDDP 75 mg/m^2^ d1 + 5-FU 1 g/m^2^/d d1-d4, Q3W/Q4W	65%	17.5		26.9%	
Zhang P, 2016 [37]	Prospective	Sq	II - IV	317	RT + CT (PF) (156)	50–70	CDDP 60 mg/m^2^ d1 + 5-FU 300 g/m^2^ d1-d3, Q4W	/	24	77.4%	32.8%	/
					RT + CT (DP) (161)	50–70	CDDP 80 mg/m^2^ d1 + Docetaxel 60 mg/m^2^ d1, Q3W; OR CDDP 25 mg/m^2^ d1 + Docetaxel 25 mg/m^2^ d1, QW	/	21			
Dashan Ai, 2022 [10]	Prospective (ESO-Shanghai2)	Sq	II - IV	321	RT + CT (TP) (107)	61.2	CDDP 25 mg/m^2^/d d1-d3 + Paclitaxel 175 mg/m^2^ d1, Q4W	/	/	81.3%	60.1%	/
					RT + CT (TF) (107)		/	/	/	79.4%	57.2%	/
					RT + CT (TC) (107)		/	/	/	79.4%	56.5%	/
Current study	Prospective	Sq	II - IV	86	RT + CT (DP) (45)	55.8–64.0	CDDP 80 mg/m^2^ d1 + Docetaxel 60 mg/m^2^ d1, Q3W	84.4%	42.27	87.3%	59.5%	52.7%
					RT + CT (PF) (41)	56.0–64.0	CDDP 80 mg/m^2^ d1 + 5-FU 1000 g/m^2^ d1-d4, Q3W	87.8%	Not reached	93.7%	65.5%	62.9%

Abbreviations: Ad = adenocarcinoma; CT = chemotherapy; DP = docetaxel + cisplatin, MST = median survival time; ORR = overall response rate; OS = overall survival; PF = cisplatin + 5-FU; QW = every week; Q3W = every three weeks; Q4W = every four weeks; Q5W = every five weeks; RT = radiotherapy; Sq = squamous cell carcinoma; TP = paclitaxel + cisplatin; TF = paclitaxel + fluorouracil; TC = paclitaxel + carboplatin; TPF = fluorouracil + cisplatin + paclitaxel; FOLFOX = oxaliplatin + leucovorin + fluorouracil.

To improve the efficacy, some studies have aimed to improve survival by optimizing the radiation dose [5, 31, 38]. Unsurprisingly, none of the results were satisfactory. And recently, an oral presentation from Maarten C.C.M. Hulshof at 2020 ASCO-GI indicated that increasing the radiotherapy dose from 50.4 to 61.6 Gy (primary tumor) did not significantly improve local control rate and OS. In contrast, the number of related side effects increased.

Along with the emergence of other drugs, several active drugs have been used in various clinical studies to obtain a better response. Phase II trial RTOG 0113, which compared non-fluorouracil-based therapy (cisplatin plus paclitaxel) and fluorouracil-based therapy (fluorouracil plus paclitaxel), reported that the median survival time were 14.9 months in the cisplatin group and 28.7 months in the fluorouracil group, respectively [34]. In PRODIGE5/ACCORD17, results showed that definitive chemoradiotherapy with FOLFOX (fluorouracil plus leucovorin and oxaliplatin) did not improve PFS compared to chemoradiotherapy with cisplatin and fluorouracil in patients with unresectable EC [3]. ESO-Shanghai 2, a randomized, multicenter, phase III clinical trial, compared three paclitaxel-based chemoradiotherapy regimens (paclitaxel with fluorouracil, carboplatin, and cisplatin) and suggested that paclitaxel plus fluorouracil was not superior to paclitaxel plus carboplatin or paclitaxel plus cisplatin in terms of OS. CCRT in patients with locally advanced ESCC, the 3-year OS rates were 57.2% in the fluorouracil group, 56.5% in the carboplatin groups, and 60.1% in the cisplatin group, respectively [10].

Docetaxel, a new generation of anti-EC drugs, enhances the radiation response by inducing apoptosis and mitotic arrest in murine tumor cells [16, 17, 39–43]. Given the known activity of cisplatin in EC, we planned to explore the combined effect of docetaxel and cisplatin as an enhanced disease control regimen for locally advanced diseases that are not resectable. Moreover, previous studies have enrolled participants with different histological types (squamous carcinoma and adenocarcinoma) or patients with different disease stages (locally advanced diseases and metastatic diseases) [44, 45]. Therefore, it is time to initiate our study, a phase II prospective randomized trial comparing the efficacy and toxicity of PF and DP regimens with CCRT in patients with ESCC. After the 5-year follow-up, we found that the ORR of the PF group was 87.3% and that of the DP group was 84.4% (P = 0.653). Besides, the 5-year OS did not differ statistically significantly between the PF group and the DP group (62.9% vs. 52.7%, p = 0.131). In the present study, OS was higher than that in previous studies. Several reasons may account for these differences between different studies. Firstly, various studies used different chemotherapy regimens with varying dose intensity. As an example, in Zhang’s study, patients received lower doses of 5-FU and cisplatin. Secondly, in our trial, chemotherapy compliance rate was substantially higher (97.6% in the PF group and 71.1% in the DP group) than that in the RTOG 8501 trial. Thirdly, there have been decades since the RTOG 8501 trial was conducted, without improvements in the techniques of radiotherapy, staging methods, or best supportive care. Finally, there may exist patient selection bias, especially in those retrospective studies, and differences between ethnicities may also play a significant role [46, 47].

At the same time, due to the time of data analysis in this article, more than half of the participants had both local recurrence and distant metastasis, coupled with the fact that a consensus salvage treatment strategy for patients with ESCC who recur after definitive chemoradiotherapy or radiotherapy has not been established. In this study, we made a summarized list of the patients who received different salvage treatments after relapse. Notably, because local progression is more common, the salvage treatments and survival of some patients with local residual or local recurrent are summarized in Table 6. The median survival time after salvage treatment was 13.5 months in these patients. The 1, 2, and 3-year OS rates were 79.0%, 50.3%, and 43.1%, respectively. In particular, three (20%) of the 15 patients remained disease free after salvage treatment. However, the optimal salvage treatment modality remains controversial (Table 6) [48–56]. Clinicians should make individualized assessments based on the type, location, and local involvement of the cancer as well as the functional status of each patient to determine the appropriate salvage treatment.

**Table 6:** Salvage therapy for esophageal cancer recurrence after definitive concurrent chemoradiotherapy

Study	Type of recurrence	N	Salvage treatment	Short-term effect	Long-term effect	Note
Makazu, 2014 [47]	local	11	sEMR	R0 rate:84.6%	5year OS 41.6%	If the lesion does not exceed the mucosal layer and there are no lymph nodes or distant metastases.
Yano, 2008 [50]	local	21	sEMR	R0 rate:84.6%	5year OS49.1%	
Takeuchi, 2013 [51]	local	19	sESD	CR rate:94.7%	3year OS 74.0%	/
Hatogai, 2016 [52]	local	113	sPDT	CR rate:58.4%	5year OS 35.9%	If the tumor invades the submucosa and sEMR treatment is unsuccessful.
Matsutani, 2017 [53]	local	12	sAPC	CR rate:58.3%	5year OS 47.0%	/
Yoo C, 2012 [54]	local	12	Salvage Esophagectomy	/	3year OS 58.0%	/
Chen Y, 2014 [55]	local	36	Salvage Chemoradiation	/	3year OS 12.2%	/
Zhou ZG, 2015 [56]	local	55	Salvage Radiotherapy	/	3year OS21.8%	/
Jie Li, 2020 [56]	distant	82	Salvage Radiotherapy	/	MST (months) RT:14 NRT:7 (P = 0.016).	Compared with BED10 < 60 Gy, BED10 ≥ 60 Gy could further prolong the median OS (16months vs. 10 months).

Abbreviations: sEMR = salvage Endoscopic mucosal resection; OS = overall survival; sESD = salvage endoscopic submucosal dissection; CR = complete response; sPDT = salvage photodynamic therapy; sAPC = salvage Argon plasma coagulation; MST = median survival time; RT = radiotherapy; NRT = non-radiotherapy; BED = biological effective dose

In summary, we were unable to confirm that the docetaxel plus cisplatin regimen performed better in OS than the fluorouracil plus cisplatin regimen in definitive CCRT for patients with ESCC. Based on these findings, our recommendation is to remain the standard role of the fluorouracil plus cisplatin regimen for definitive CCRT in patients with locally advanced ESCC. What’s more, comparing the adverse event (AE) profiles of the two regimens, it was found that the docetaxel plus cisplatin regimen resulted in severe leukocytopenia and neutropenia, and that the treatment had to be interrupted or terminated. As for late adverse effects, there was no significant difference between two groups. One possible reason is the difficulty in collecting late effects data.

In view of the biological characteristics of ESCC, chemotherapy has entered a bottleneck stage; however, it still plays an irreplaceable role in CCRT. Recent studies have indicated that, in combination with immunotherapy, chemoradiotherapy has shown immunomodulatory effects that may result in synergistic treatment responses. Some ongoing studies have explored the combination of chemoradiotherapy regimens with immunotherapies in EC, such as KEYNOTE-975 (study of pembrolizumab in combination with chemoradiotherapy in EC), ESOCORT-CRT (study of camrelizumab (SHR-1210) in combination with concurrent chemoradiotherapy in EC), and Rational-311 (study of Tislelizumab [BGB-A317] versus placebo in combination with chemoradiotherapy in EC), and we expect to hear good news from these studies in the near future.

## Conclusions

The 5-year follow-up confirmed that definitive CCRT with the DP regimen did not improve the treatment response, OS, or PFS in patients with ESCC compared to the PF regimen. The PF regimen remains the standard regimen for definitive CCRT for patients with locally advanced ESCC. Long-term follow-up also suggested that appropriate and active salvage treatment has a survival benefit for some patients, and late cardiopulmonary adverse event should be noticed during follow-up.

## Data Availability

The datasets used and/or analyzed during the current study are available from the corresponding author on reasonable request.

## Abbreviations

DP: docetaxel and cisplatin
PF: cisplatin and 5-fluorouracil
CCRT: concurrent chemoradiotherapy
ESCC: esophageal squamous cell carcinoma
OS: overall survival
PFS: progression free survival
EC: esophageal carcinoma
ORR: overall response rate
CR: complete remission
PR: partial remission
SYSUCC: Sun Yat-sen University Cancer Center
KPS: Karnofsky performance status
AJCC: American Joint Committee on Cancer
CT: computed tomography
PET/CT: positron emission tomography
GTV: gross tumor volume
IFI: involved field irradiation
CTV: clinical target volume
PTV: planning target volume
IMRT: intensity-modulated radiation therapy
3D-CRT: three-dimensional conformal radiotherapy
CTCAE 3.0: Common Terminology Criteria for Adverse Events version 3.0
FOLFOX: fluorouracil plus leucovorin and oxaliplatin
AE: adverse event
BMI: body mass index
CCI: Charlson comorbidity index
SD: stable disease
PD: progressive disease
FOLFIRI: 5-fluorouracil + irinotecan + leucovorin
anti-PD-1: anti-program death-1
Ad: adenocarcinoma
MST: median survival time
QW: every week
Q3W: every three weeks
Q4W: every four weeks
Q5W: every five weeks
RT: radiotherapy
Sq: squamous cell carcinoma
TP: paclitaxel + cisplatin
TF: paclitaxel + fluorouracil
TC: paclitaxel + carboplatin
TPF: fluorouracil + cisplatin + paclitaxel
sEMR: salvage Endoscopic mucosal resection
sESD: salvage endoscopic submucosal dissection
sPDT: salvage photodynamic therapy
sAPC: salvage Argon plasma coagulation
NRT: non-radiotherapy
BED: biological effective dose
CDDP: cisplatin
DOC: docetaxel
Fr: fraction
